# Reflective, polarizing, and magnetically soft amorphous neutron optics with ^11^B-enriched B_4_C

**DOI:** 10.1126/sciadv.adl0402

**Published:** 2024-02-14

**Authors:** Anton Zubayer, Naureen Ghafoor, Kristbjörg Anna Thórarinsdóttir, Sjoerd Stendahl, Artur Glavic, Jochen Stahn, Gyula Nagy, Grzegorz Greczynski, Matthias Schwartzkopf, Arnaud Le Febvrier, Per Eklund, Jens Birch, Fridrik Magnus, Fredrik Eriksson

**Affiliations:** ^1^ Thin Film Physics Division, Department of Physics, Chemistry and Biology (IFM), Linköping University, SE-581 83 Linköping, Sweden.; ^2^ Science Institute, University of Iceland, Dunhaga 3, IS-107 Reykjavik, Iceland.; ^3^ Paul Scherrer Institut, 5232 Villigen PSI, Switzerland.; ^4^ Department of Physics and Astronomy, Uppsala University, SE-75120, Uppsala, Sweden.; ^5^ Photon Science, DESY, Notkestraße 85, 22607 Hamburg, Germany.

## Abstract

The utilization of polarized neutrons is of great importance in scientific disciplines spanning materials science, physics, biology, and chemistry. However, state-of-the-art multilayer polarizing neutron optics have limitations, particularly low specular reflectivity and polarization at higher scattering vectors/angles, and the requirement of high external magnetic fields to saturate the polarizer magnetization. Here, we show that, by incorporating ^11^B_4_C into Fe/Si multilayers, amorphization and smooth interfaces can be achieved, yielding higher neutron reflectivity, less diffuse scattering, and higher polarization. Magnetic coercivity is eliminated, and magnetic saturation can be reached at low external fields (>2 militesla). This approach offers prospects for substantial improvement in polarizing neutron optics with nonintrusive positioning of the polarizer, enhanced flux, increased data accuracy, and further polarizing/analyzing methods at neutron scattering facilities.

## Teaser

^11^B_4_C integration increases reflectivity and polarization, decreases diffuse scattering, and makes magnetically soft multilayers.

## INTRODUCTION

The importance of polarized neutrons at the forefront of today’s materials science, physics, chemistry, and biology is underscored by their broad applicability for characterization of thin films, nanostructures, powders, liquids, and crystals. Polarization analysis offers insights into otherwise unattainable sample information such as magnetic domains (*1*) and structures (*2*); protein crystallography (*3*); composition, orientation, and ion-diffusion mechanisms (*4*); and relative location of molecules in multicomponent biological systems (*5*). Nevertheless, inherent limitations to state-of-the-art neutron optics limit their broad applicability. Specifically, there is a need for improvement in four key areas: reflective capabilities (higher intensity and reflectivity at higher scattering vectors/angles), polarization (enhanced polarization at low scattering angles/vectors and the possibility for polarization at higher scattering angles/vectors), reduction of magnetic coercivity, and reduced diffuse scattering. Here, we address all four of these challenges by incorporation of low-neutron-absorbing isotope-enriched ^11^B_4_C in Fe/Si-polarizing multilayer neutron optics deposited by dc magnetron sputtering.

First, to enhance reflectivity, it is necessary to reduce the interface width between the layers in the multilayer structure. Now, the interface (and/or iron silicide layer) width of Fe/Si-polarizing multilayer neutron optics is usually around 8 to 11 Å (*6*). By preventing silicide formation and minimizing roughness, the reflectivity can be substantially increased. In addition, thinner layers can be deposited, allowing reflection at higher scattering vectors.

Second, achieving high polarization requires a suitable contrast or match between the nuclear and magnetic scattering length densities (SLDs) of the magnetic and nonmagnetic layers, depending on the spin state. The sum of the nuclear SLD (nSLD) and the magnetic SLD (mSLD) of the magnetic layer should exhibit high contrast with the SLD of the nonmagnetic layer. Conversely, the difference between the nSLD and the mSLD of the magnetic layer should match the SLD of the nonmagnetic layer. This arrangement ensures that spin-up neutrons are reflected while spin-down neutrons are not, thereby achieving high polarization (*7*).

Third, Fe/Si multilayers typically exhibit a crystalline structure, leading to the presence of magnetic domains and a high magnetic coercivity, which is typically associated with magnetic particle/grain size (*8*). The high coercivity can be addressed by the use of magnetically soft materials, i.e., materials that can be easily magnetized and demagnetized, yielding low magnetic coercivity. For example, the use of soft Fe can reduce the magnetic coercivity by several orders of magnitude (*9*). By amorphizing the Fe layers (*10*), a smaller external field would be required to saturate the magnetization, allowing the optics to be positioned very close to the sample environment to enable polarization without magnetic stray fields affecting the sample environment itself.

Fourth, it is crucial to minimize and characterize the diffuse scattering from neutron optical elements to be able to deduce whether the scattered neutrons come from the polarizer or the sample of interest. Because diffuse scattering from multilayer optics originates from interfacial roughness and nanocrystallites, it will be largely reduced by amorphization and formation of flatter interfaces. We examine the diffuse scattering outside the specular view so that lateral correlations (*11*) in the multilayers can be quantified.

**Fig. 1.**
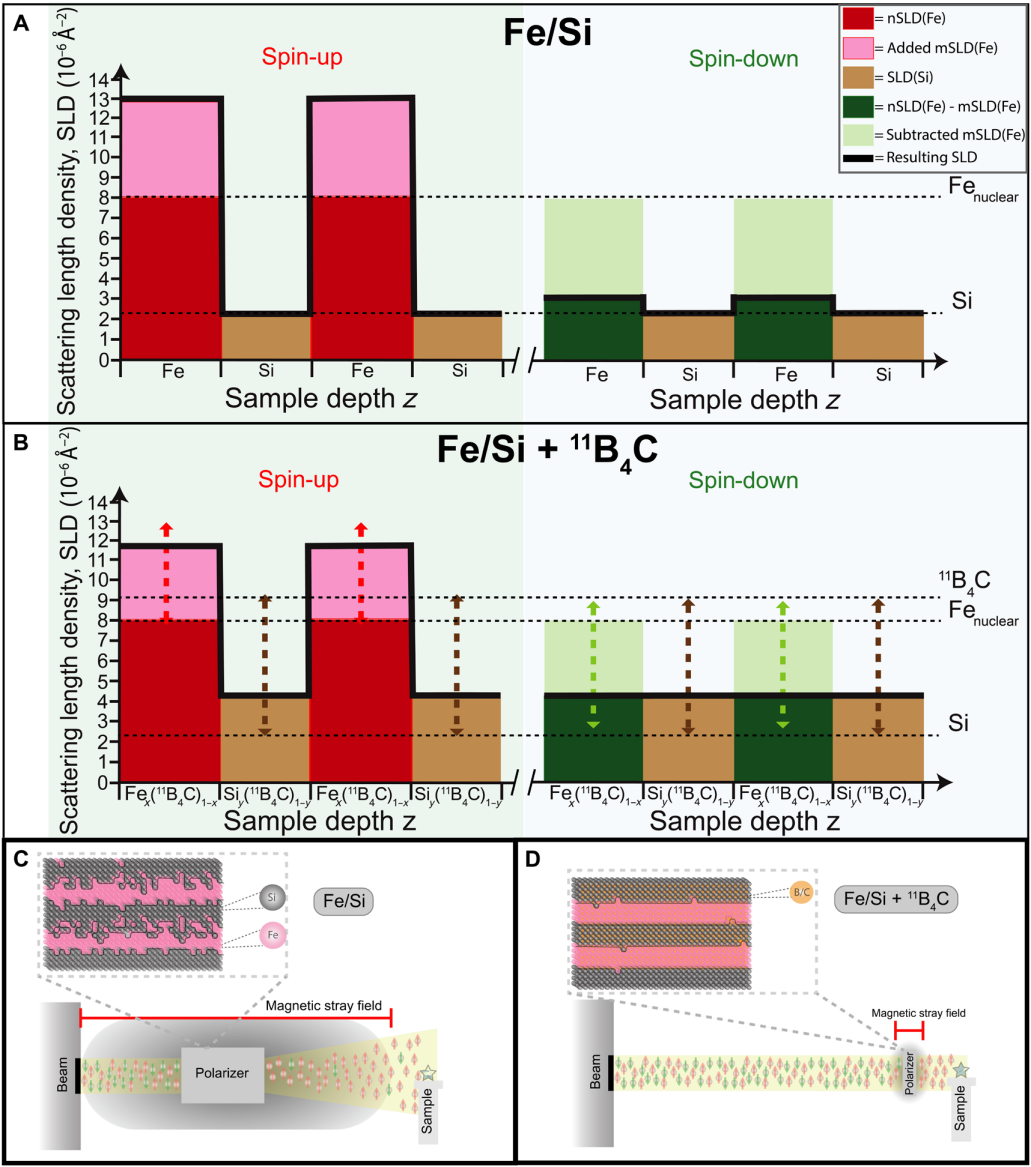
Concept of SLD tuning and implementation of improved polarizing neutron optics. (**A** and **B**) Illustrations showing the SLD depth profiles (solid black curves) resulting from the different magnetic and nuclear SLDs (mSLDs and nSLDs) for (A) Fe/Si and (B) ^11^B_4_C-containing Fe/Si multilayers. In the spin-up configuration, the mSLD of Fe is added to the nSLD of Fe, while, in the spin-down configuration, the mSLD of Fe is subtracted from the nuclear SLD of Fe. The dashed arrows in (B) represent the SLD tuning possibilities achieved by incorporating varying amounts of ^11^B_4_C. (**C** and **D**) Comparison of Fe/Si (C) with Fe/Si + ^11^B_4_C (D) highlighting the advantage of using a lower applied magnetic field for polarizer saturation. This, in turn, allows for nonintrusive positioning of the polarizer closer to the sample environment, which can be achieved by incorporating ^11^B_4_C to eliminate magnetic coercivity, in other words, make the polarizer magnetically soft.

The incorporation of isotope-enriched ^11^B_4_C presents an opportunity to address these issues. ^11^B_4_C, instead of just ^11^B, is used because ^11^B_4_C sputter targets are more stable, cheaper, and available. First, ^11^B_4_C could reduce interface roughness (*12*) through elimination of crystalline grain growth by amorphization and prevent silicide formation through the strong bonds between B and Fe. Second, the high SLD of ^11^B_4_C allows for tuning the SLDs. Third, by eliminating lateral correlations, diffuse scattering can be minimized. Fourth, amorphization also eliminates magnetic coercivity. The amorphization is due to the stronger Fe-B bonds than Fe-Fe or Fe-Si bonds, where the enthalpies of formations are −38 and −26 kJ/mol for Fe-B and Fe-Si, respectively (*13*). Consequently, preventing the formation of iron silicides and crystalline Fe leads to a reduction in interface width. The concept of SLD tuning using different amounts of ^11^B_4_C incorporated in the Fe and Si layers is illustrated in Fig. 1 (A and B).

**Fig. 2.**
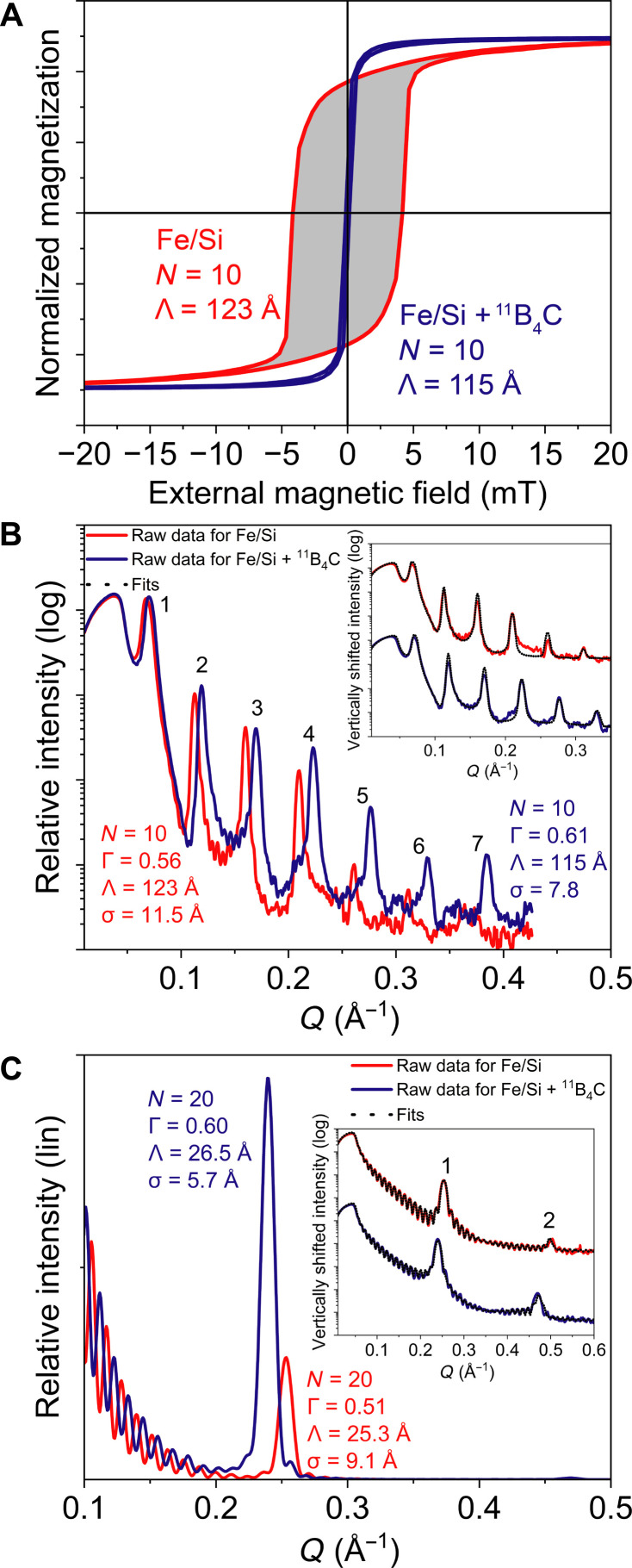
Magnetism and interface improvement. (**A**) Hysteresis curves showing the normalized magnetization measured by vibrating sample magnetometry (VSM) for Fe/Si and Fe/Si + ^11^B_4_C multilayers. The gray area represents the magnetic energy that is dissipated during the magnetization reversal process within the material, which is eliminated in the case of Fe/Si + ^11^B_4_C multilayers. (**B**) X-ray reflectivity (XRR) data of an Fe/Si and an Fe/Si + ^11^B_4_C multilayer, where the inset shows the XRR fits of the data seen in (B). (**C**) XRR data, in linear scale, of an Fe/Si and an Fe/Si + ^11^B_4_C multilayer sample. The inset shows XRR fits of the data seen in (C). The fitting parameters showed that, in the Fe/Si case, the layers had a 13-Å Fe layer and a 13-Å Fe-and-Si mixed layer, indicating the absence of pure Si layers. In contrast, for Fe/Si + ^11^B_4_C, a 5.7-Å interface width was observed.

Figure 1 (A and B) illustrates the SLD depth profiles for spin-up and spin-down configurations for Fe/Si (Fig. 2A) and Fe/Si + ^11^B_4_C (Fig. 2B), i.e., with ^11^B_4_C incorporated in both the Fe and the Si layers in varying amounts.

The goal of polarization is to maximize contrast for spin-up and minimize it for spin-down. The Fe/Si configuration, with some contrast in spin-down, can be improved by adding ^11^B_4_C to the Si layer to increase its SLD. However, diluting Fe with ^11^B_4_C decreases the mSLD of Fe, which can be compensated by further increasing the concentration of ^11^B_4_C in Si. The dashed arrows indicate the range of tunability via ^11^B_4_C incorporation at different concentrations. SLD tuning has been attempted using ion-beam sputtering and Ar implantation during Fe/Ge multilayer growth (*14*) and by the addition of N_2_- and O_2_-reactive gases during growth of Fe/Si supermirrors (*15*). However, these methods were limited by attainable SLDs and challenges in trapping gas elements. The current approach allows larger SLD tuning (from 2.1 × 10^−6^ to 9.0 × 10^−6^ Å^−2^), which is much wider than previous methods, leading to maximum polarization and precise layer SLD adjustment in a thin film. A comparison between Fe/Si (Fig. 1C) with Fe/Si + ^11^B_4_C (Fig. 1D) indicates the effect of a reduction in magnetic stray field owing to a lower applied field needed to reach magnetic saturation for a magnetically soft polarizer due to amorphization of Fe (*10*), in accordance with the results presented in this work. In addition, a comparison of the two configurations illustrates the decrease in diffuse scattering for Fe/Si + ^11^B_4_C, attributed to the reduction of lateral correlations that give rise to the diffuse scattering in pure Fe/Si.

Samples of Fe/Si and Fe/Si + ^11^B_4_C were grown using magnetron sputter deposition and were studied using x-ray diffraction (XRD), x-ray reflectivity (XRR), grazing incidence small-angle x-ray scattering (GISAXS) and grazing incidence wide-angle scattering (GIWAXS), elastic recoil detection analysis (ERDA), polarized neutron reflectivity (PNR), vibrating sample magnetometry (VSM), transmission electron microscopy (TEM), and x-ray photoelectron spectroscopy (XPS). Throughout this study, we refer to *N* as the number of bilayers in the multilayer; Γ as the layer thickness ratio being the thickness of the Fe layer divided by the bilayer thickness [i.e., Fe layer/(Fe layer + Si layer)]; and Λ as the bilayer thickness.

## RESULTS

An initial series of experiments was performed to determine suitable concentrations of ^11^B_4_C and multilayer periods, as detailed in section S1. On the basis of these initial experiments, a content of 14 vol % ^11^B_4_C was chosen for both Fe and Si layers to maintain the Fe layer in an x-ray amorphous state. The magnetic properties of Fe/Si and Fe/Si + ^11^B_4_C with 14 vol % ^11^B_4_C in the Fe and Si layers were studied using VSM. The results, presented in Fig. 2A, demonstrate that the inclusion of 14 vol % of ^11^B_4_C completely eliminated the coercivity, resulting in a fivefold reduction in the external magnetic field required for magnetization saturation. The Fe/Si sample required ~10 mT for saturation, whereas Fe/Si + ^11^B_4_C only required ~2 mT. Figure 2B displays the XRR measurements and fits for Fe/Si and Fe/Si + ^11^B_4_C multilayers with a nominal bilayer thickness of 100 Å and nominal thickness ratio of 0.5. The higher Bragg peak intensities, numbered 1 to 7, for the Fe/Si + ^11^B_4_C sample are indicative of a decreased interface width compared to Fe/Si multilayer. Furthermore, fits of the XRR measurements for Fe/Si and Fe/Si + ^11^B_4_C multilayers with a nominal bilayer thickness of 25 Å in Fig. 2C indicate that the Fe/Si sample, although there is a chemical modulation, exhibits extensive mixing of Fe and Si throughout the entire sample, likely due to the expected formation of iron silicide. In contrast, the addition of ^11^B_4_C prevents the formation of iron silicide and other crystalline phases, as indicated by fig. S1A. The interface width, obtained through the fitting, for Fe/Si + ^11^B_4_C was 5.7 Å, and, thus, the incorporation of ^11^B_4_C leads to more distinct separation between magnetic and nonmagnetic layers and reduction in interface width. Regardless of bilayer thickness, the inclusion of ^11^B_4_C resulted in decreased interface width and substantially increased reflectivity.

An extended range of samples was grown using ion-assisted magnetron sputter deposition in a different chamber and characterized (see section S1). The results obtained from this extended study corroborate the presented findings and confirm the robustness and reproducibility of the observed effects.

**Fig. 3.**
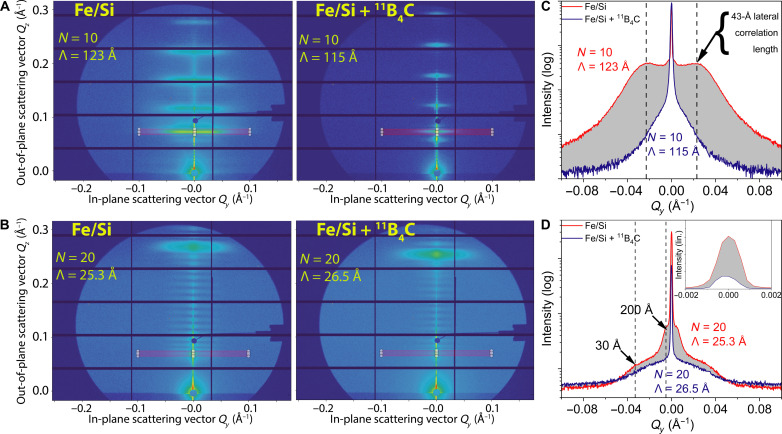
Lateral correlation and diffuse scattering analysis. Grazing incidence small-angle x-ray scattering (GISAXS) measurements were performed on Fe/Si and Fe/Si + ^11^B_4_C samples. (**A** and **B**) The raw GISAXS maps for Fe/Si and Fe/Si + ^11^B_4_C, respectively, for bilayer thicknesses of Λ = 100 and 25 Å and number of bilayers *N* = 10 and 20, respectively, and a layer thickness ratio of Γ = 0.5. The red rectangles highlight the first off-specular Bragg sheet. (**C** and **D**) The horizontal line scan of the first Bragg sheet of Fe/Si (black) and Fe/Si + ^11^B_4_C (red) of 100 Å and *N* = 10 and 25 Å and *N* = 20, respectively, and a layer thickness ratio of Γ = 0.5, derived from the GISAXS raw data seen in (A) and (B). The inset in (D) shows the intensity of the Bragg sheet in the *Q_y_* direction on a linear scale.

To investigate the off-specular/diffuse scattering and the lateral correlations (*11*, *16*, *17*), GISAXS was used. The constructive interference of repeating surface and interface features in the sample were examined to assess the lateral correlations in the in-plane direction. Figure 3 (A and B) presents the raw GISAXS data obtained from multilayers of Fe/Si (Fig. 3A) and Fe/Si + ^11^B_4_C (Fig. 3B) for nominal bilayer thicknesses Λ and number of bilayers *N* being Λ = 100 Å and *N* = 10 in Fig. 3A and Λ = 25 Å and *N* = 20 in Fig. 3B. The results indicate a substantial reduction in off-specular scattering in the Fe/Si + ^11^B_4_C sample compared to the Fe/Si sample, highlighting the impact of ^11^B_4_C incorporation on mitigating lateral correlations. To quantitatively deduce the lateral correlations causing the off-specular scattering, line scans were taken of the first Bragg sheet, as indicated by the red rectangles in Fig. 3 (A and B). These line scans show the intensity distribution in the *Q_y_* direction. The gray area between the black (Fe/Si) and red (Fe/Si + ^11^B_4_C) lines in Fig. 3 (C and D) represents the reduction in diffusely scattered intensity at the first Bragg sheet in the Fe/Si sample compared to the Fe/Si + ^11^B_4_C sample, showing a substantial reduction in diffuse scattering in the presence of ^11^B_4_C.

The Fe/Si samples, seen in Fig. 3 (C and D), exhibits shoulders, corresponding to constructive interference of repeating features in the lateral direction. The obtained lateral correlation length is ~43 Å, as determined from the position of the shoulders in Fig. 3C. However, when ^11^B_4_C is incorporated, these periodic correlations are eliminated. This can be attributed to the elimination or substantial reduction in crystallite formation caused by the incorporation of ^11^B_4_C. Hence, the lateral correlations associated with crystalline Fe or iron silicides are also diminished as seen in Fig. 3. In Fig. 3D, the line scan of the Fe/Si + ^11^B_4_C samples with Λ = 25 Å and *N* = 20 is shown, and it is observed that the incorporation of ^11^B_4_C reduces the off-specular scattering and eliminates the shoulders. The presence of shoulders in the Fe/Si sample suggests the presence of larger hills with smaller hills on top in the lateral directions, with lateral correlation lengths of ~200 and 30 Å. The inset in Fig. 3D shows the linear intensity of the off-specular scattering in the *Q_y_* direction. It clearly illustrates that the Fe/Si sample exhibits a higher off-specular intensity compared to Fe/Si + ^11^B_4_C. This further emphasizes the importance of incorporating ^11^B_4_C to reduce the diffuse scattering of the reflected beam and improve the overall sample characteristics.

**Fig. 4.**
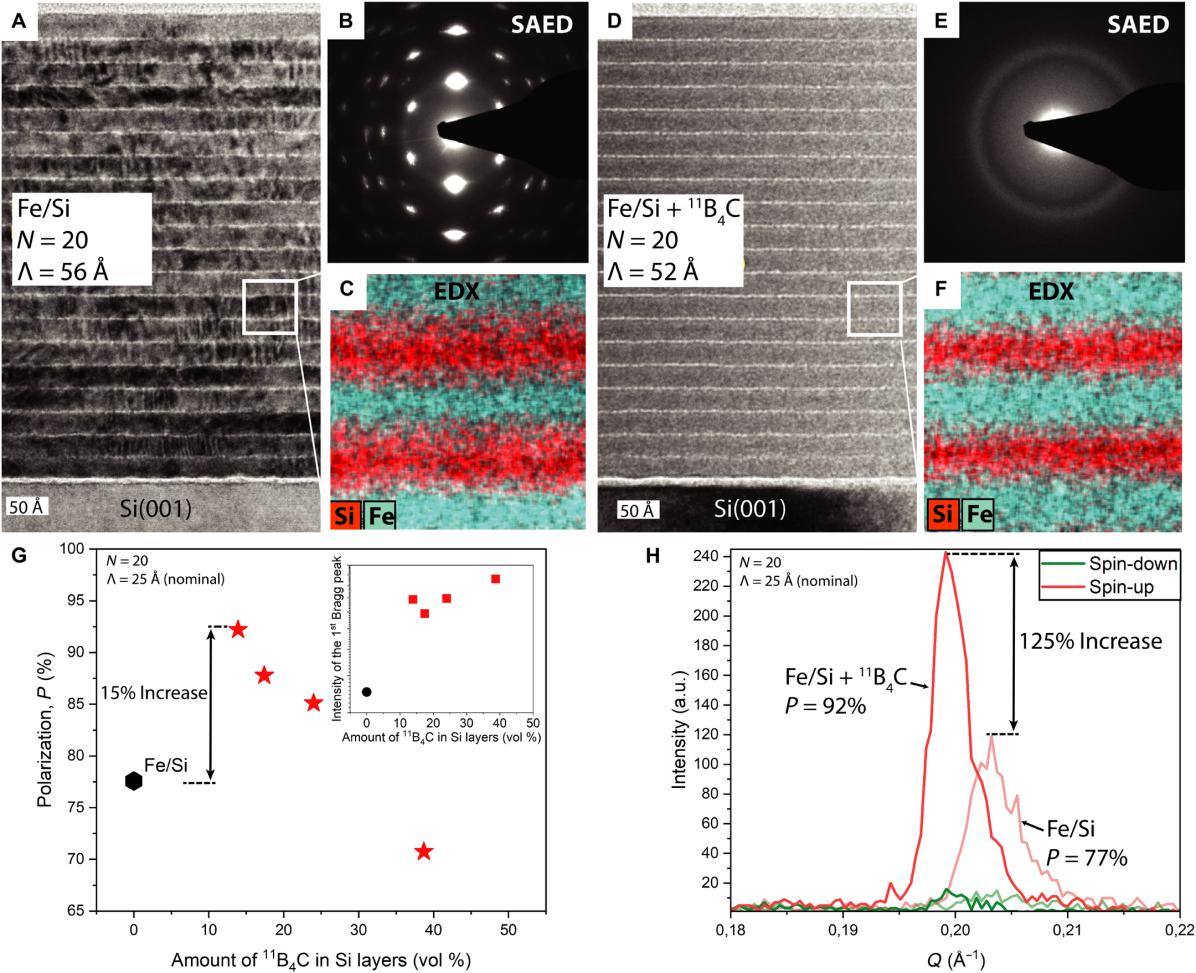
Microscopy, polarization, and reflectivity. (**A** to **C**) Overview diffraction contrast cross-sectional TEM micrograph, corresponding selected-area electron diffraction (SAED) pattern, and colored coded (Fe-cyan, Si-red) 135 Å–by–135 Å energy-dispersive x-ray (EDX) elemental map of Fe/Si multilayer, respectively. (**D** to **F**) Overview diffraction contrast TEM micrographs, corresponding SAED pattern, and colored coded (Fe-green, Si-red) 135 Å–by–135 Å EDX elemental map of Fe/Si + ^11^B_4_C multilayer with 14 vol % of ^11^B_4_C, respectively. Both multilayers have bilayer thickness of Λ = 50 Å and *N* = 20 number of bilayers. (**G**) Polarization (*P*) at the Bragg peak for four samples, indicated with red stars, with different ratios between ^11^B_4_C and Si. The polarization of the Fe/Si sample is represented by the black hexagon. The inset shows the intensity of the first Bragg peak for the spin-up case for these samples. (**H**) PNR in the *Q* range between 0.18 and 0.22 Å^−1^ to visualize the first Bragg peak for Fe/Si and the best polarizing Fe/Si + ^11^B_4_C sample. The polarization values are obtained through 10-min measurements at the specific Bragg peak position. a.u., arbitrary units.

Comparing TEM and electron diffraction analyses, as seen in Fig. 4 (A and B) for Fe/Si and Fig. 4 (D and E) for Fe/Si + ^11^B_4_C, suggests that adding ^11^B_4_C prevents iron silicide formation and leads to an amorphous multilayer, also in good agreement with XRD in fig. S1A. GIWAXS measurements in fig. S4 also support this, showing the 14 vol % ^11^B_4_C sample as nearly fully amorphous, while Fe/Si shows clear crystallinity. The images in Fig. 4 (A and D) appear as if the Fe layer is very thick compared to the Si layer, then this could be due to an instrumental artifact. Note that the Fe and Si layers are not clearly separated, and there seems always to be a gradient of Fe and Si content throughout the multilayer for both samples. However, comparing the energy-dispersive x-ray maps of Fig. 4 (C and F), the intermixing between Fe and Si atoms is much smaller when ^11^B_4_C is incorporated.

PNR measurements, as seen in Fig. 4G, aim not only to validate enhanced reflectivity, polarization, and SLD tunability but also to demonstrate these achievements at higher scattering angles/vectors than typical polarizing neutron optics. State-of-the-art commercial optics can reach reflectivity up to *Q* = 0.119 Å^−1^ (*18*). PNR measurements show better polarization for certain ^11^B_4_C concentrations in Si compared to that in Fe/Si. The optimal sample provides 15% higher polarization and 125% higher reflectivity, demonstrating the benefits of ^11^B_4_C in SLD-matching and for reducing interface width for higher reflectivity. To explore the impact for thicker layers, reflectivity and polarization were simulated using the parameters obtained from the reflectivity fits in Fig. 2B for Λ = 200 Å and *N* = 7 (section S5). These results showed that the reflectivity difference is not as pronounced for thicker bilayers as for thinner ones, as seen in Fig. 4D, and the polarization increase is only 1% compared to the 15% observed for thinner bilayers.

## DISCUSSION

In summary, this study has addressed the four main outstanding issues concerning polarizing multilayer neutron optics. First, the incorporation of ^11^B_4_C in Fe/Si multilayers leads to higher reflectivity, which can enhance the neutron flux. Second, the improved polarization through SLD tuning with the inclusion of ^11^B_4_C enables experiments that require high polarization sensitivity. Third, the reduction in diffuse scattering in the polarizer is beneficial for experiments involving the study of diffuse scattering from the sample. Last, the elimination of coercivity allows for the polarizer to be positioned closer to the sample environment. This yields greatly improved reflectivity and polarization and offers prospects for experiments with nonintrusive positioning of the polarizer/analyzer near the sample environment with enhanced flux, data accuracy, and polarizing/analyzing methods in neutron scattering facilities.

## MATERIALS AND METHODS

Fe/Si and Fe/Si + ^11^B_4_C multilayer thin films were deposited using dc magnetron sputter deposition in an ultrahigh vacuum system with a background pressure of about 2.6 × 10^−7^ Pa (2 × 10^−9^ torr). Further details of the deposition system can be found elsewhere (*19*). The multilayers were deposited on 10 mm–by–10 mm–by–0.5 mm Si(100) substrates with a native oxide. The substrate was kept under constant rotation of 15 rpm and at room temperature. Fifty-millimeter targets of Fe (Plasmaterials, 99.95% purity), ^11^B_4_C (RHP Technology, 99.8% chemical purity, >90% isotopic purity), and Si (Kurt J. Lesker, 99.95% purity) were used and powered at 33, 50, and 10 to 40 W, respectively. The sputtering rates were determined from XRR thickness measurements of multilayers. The multilayers were achieved by using computer-controlled shutters where the opening durations were selected for the desired layer thicknesses and design of the multilayer. During the deposition, a substrate bias of −30 V was applied. When depositing Fe/Si + ^11^B_4_C, each bilayer consisted of co-sputtered ^11^B_4_C with Fe, followed by the deposition of ^11^B_4_C with Si. The deposition rates of both Fe + ^11^B_4_C and Si + ^11^B_4_C were nearly equal, ~0.5 Å/s. When preparing samples with different ratios of Si to ^11^B_4_C, only the deposition rate of Si was adjusted by tuning its target power.

Hard XRR analysis was carried out using an Empyrean diffractometer by Panalytical in a parallel beam geometry with a line-focused copper anode source operating at 45 kV and 40 mA giving Cu-K_a_ radiation with a wavelength of 1.54 Å. In the incident beam path, a parabolic x-ray mirror was used along with a ^1^/_2_° divergence slit to condition the beam and limit the x-ray spot size on the sample. In the diffracted beam path, a parallel plate collimator with a parallel plate collimator slit (0.27°) was used, followed by a PIXcel detector operating in open detector mode.

XRD was performed using a Panalytical X’Pert diffractometer in Bragg-Brentano geometry with a Bragg-Brentano HD incident beam optics module with a ^1^/_2_° divergence slit and a ^1^/_2_° anti-scatter slit. On the secondary optics side, a 5-mm anti-scatter slit was used together with an X’celerator detector operating in scanning line mode. Diffraction measurements were performed using a 2θ scanning range of 20° to 90°, a step size of 0.018°, and a collection time of 20 s.

To analyze the XRR data, the multilayer bilayer thickness and interface roughness were determined through fitting. The fitting process involved the software GenX (v3) (*20*), which enabled the simulation of the ratio between ^11^B_4_C and Si in the nonmagnetic layer to achieve the necessary SLD match between the magnetic and nonmagnetic materials. In addition, GenX was used to fit the retrieved data from corresponding x-ray and neutron reflectivity measurements.

The magnetic properties of the samples were analyzed using VSM in a longitudinal geometry at room temperature. Magnetic hysteresis curves were measured in a field range of −20 to 20 mT, providing information on the saturation magnetization and coercive field of the samples.

Time-of-flight ERDA (ToF-ERDA) at the Tandem Laboratory of Uppsala University (*21*) was used to determine the elemental composition of selected samples. For the ToF-ERDA measurements, a beam of 36-MeV I^8+^ ions was used at an incident angle of 67.5° with respect to the surface normal, and the ToF and energy detectors were placed at a 45° angle with respect to the incident beam direction. The Potku code (*22*) was used for data analysis. ToF-ERDA is a depth resolved and quantitative technique to determine sample composition, without any matrix effects or need to use reference standards. In this case, the depth resolution is not sufficient to distinguish the individual layers in the multilayer samples, which are on the order of a few angstroms. Therefore, the simulated and calculated composition is an effective value, averaged over the layers.

The PNR experiments were conducted using the polarized neutron reflectometer MORPHEUS, located at SINQ (Paul Scherrer Institut, Villigen, Switzerland). In PNR experiments, a non-polarized neutron beam initially encounters the polarizer, which polarizes the beam to allow only one spin orientation to pass through. The spin state (up or down) can be switched by a spin flipper. Subsequently, the polarized neutron beam is directed at the sample surface at a small angle (θ) and undergoes specular reflection. The reflected intensity is determined by the reflection and transmission at all interfaces. PNR provides sensitivity to the spin-dependent SLD of the sample, enabling the investigation of its magnetic properties. The two possible spin orientations result in to two distinct PNR reflectivity curves. The observed Bragg peaks arise from constructive interference. The samples were measured in an external magnetic field that was sufficiently strong to magnetically saturate them in the in-plane direction, with an approximate field strength of 20 mT at the sample environment. The measurements were carried out from 0° to 15° in 2θ using a wavelength of 4.83 Å.

The preparation of cross-sectional TEM samples involved mechanical grinding and polishing, followed by Ar-ion beam milling until electron transparency was achieved. The FEI Tecnai G2 TF 20 UT microscope operating at 200 kV was used to conduct TEM.

The XPS was used to analyze the effect of adding ^11^B_4_C to the Fe/Si multilayers on the Fe-Si bond formation. The equipment used was Kratos Axis Ultra DLD instrument with a monochromatic Al Kα radiation of 1486.6 eV. The pass energy was set to 160 eV for the survey scans and to 20 eV for higher energy resolution scans. In the latter case a full width at half maximum of the Ag 3d_5/2_ peak from reference sample was 0.55 eV. Spectrometer was calibrated by measuring Au 4f_7/2_, Ag 3d_5/2_, and Cu 2p_3/2_ peak positions from sputter-etched Au, Ag, and Cu samples and comparing obtained values to the recommended ISO standards for monochromatic Al Ka sources (*23*). The base pressure during XPS analyses was better than 1.1 × 10^−9^ torr (1.5 × 10^−7^ Pa). The Fe 2p, Si 2p, O 1s, B 1s, and C 1s core level spectra were recorded. Sputter depth profiles were done using 0.5 keV Ar^+^ ion beam incident at an angle of 20^o^ from the surface and rastered over an area of 3 mm by 3 mm. All spectra are charge referenced to the Fermi level (*24*). The scanning area from where the spectra were collected was 0.3 mm by 0.7 mm centered in the middle of the etched area. Two samples were studied by XPS: Fe/Si and Fe/Si + ^11^B_4_C multilayers, both grown on 15 mm–by–15 mm Si substrates. Both multilayers had 10 bilayers/periods and a bilayer thickness of 100 Å, while the thickness ratio in each bilayer was 1:1 (Fe:Si) nominally. Spectra were taken from within the Si layer.

We conducted GISAXS experiments using the Micro- and Nanofocus X-ray Scattering beamline (MiNaXS/P03) located at the PETRA III third-generation synchrotron source of the Deutsches Elektronen-Synchrotron (DESY) in Hamburg, Germany (*25*). A beam of 32 μm–by–27 μm (horizontal by vertical) shape with a monochromatic x-ray energy of 13 keV was used. For our detector, we used a PILATUS 2 M from Dectris in Switzerland, which had a resolution of 1475 × 1679 pixels (horizontal by vertical) with a pixel size of 172 μm in both the horizontal and vertical directions. The sample-to-detector distance was set at 2470 mm. To prevent the detector from being saturated, we masked both the direct and specular reflected beam using two separate point-like beam stops. We selected α_i_ = 0.45° as the angle of incidence in the GISAXS geometry, which was greater than all critical angles of the materials involved.

## Supplementary Materials

### This PDF file includes:

Sections S1 to S7 Figs. S1 to S5 Tables S1 to S3
