# Supplementary material for: Reflective, polarizing, and magnetically soft amorphous neutron optics with 11B-enriched B4C
Source: Sci Adv. 2024 Feb 14;10(7):eadl0402. doi: 10.1126/sciadv.adl0402 (PMC10866559; doi:10.1126/sciadv.adl0402)
Supplement: Supplementary file 1 — Sections S1 to S7 Figs. S1 to S5 Tables S1 to S3 [file sciadv.adl0402_sm.pdf]

Science Advances
AAAS

Supplementary Materials for

Reflective, polarizing, and magnetically soft amorphous neutron optics with ^{11}B-enriched B_4C

Anton Zubayer *et al.*

Corresponding author: Anton Zubayer, anton.zubayer@liu.se; Per Eklund, per.eklund@liu.se; Jens Birch, jens.birch@liu.se; Fridrik Magnus, fridrikm@hi.is; Fredrik Eriksson, fredrik.eriksson@liu.se

Sci. Adv. **10**, eadl0402 (2024)
DOI: 10.1126/sciadv.adl0402

This PDF file includes:

Sections S1 to S7
Figs. S1 to S5
Tables S1 to S3

Supporting Information

Section S1. Determination of $^{11}B_4C$ content and amorphization conditions

To investigate the effects of $^{11}B_4C$ on Fe-layer amorphization, roughness and formation of iron silicide, X-ray diffraction (XRD) analysis was performed of samples synthesized with different concentrations of $^{11}B_4C$. These samples were grown in a different sputtering chamber, see section S7. The crystalline nature of Fe/Si multilayers for 0 vol.%, 2.5 vol.% and 5 vol.% is indicated by the presence of Fe (110) and/or Fe_3Si (220) peaks that combine to form a broad diffraction peak around 45° 2θ. The width of this peak is a result of the presence of grains with varying sizes.[7] 14 vol.% on the other hand was enough to yield X-ray amorphous multilayers, free from Fe or Fe-silicide crystallites. It has been established that amorphous ferromagnets exhibit substantially lower coercivity than their crystalline counterparts. In order to verify this statement, the magnetic properties of an Fe/Si sample and an Fe/Si + $^{11}B_4C$ sample with 14 vol.% $^{11}B_4C$ within the Fe and Si layers were studied using vibrating sample magnetometry (VSM). The results, depicted in Fig. S1B, reveal that the inclusion of 14 vol.% of $^{11}B_4C$ indeed eliminated the coercivity, and resulted in a difference of more than an order of magnitude in the external magnetic field required to saturate the magnetization. To saturate the Fe/Si at least 10 mT were needed while Fe/Si + $^{11}B_4C$ barely needed 1 mT. It Is important to note that the 14 vol.% lower Fe content in the $^{11}B_4C$ incorporated sample leads to a decrease in the total magnetization, which can be observed in the amplitude drop in the $^{11}B_4C$ incorporated sample. The slightly lower magnetization affects the m-SLD by decreasing it with the same ratio[8] but could, through our concept, easily be compensated for by increasing the non-magnetic layer SLD to achieve optimal polarization (Fig. 1B) which was then also done as seen in Fig. 4G.

The effect on the reflective properties for various concentrations of $^{11}B_4C$ within the Fe/Si multilayers can be seen in Fig. S1C where the Bragg peak intensity increases with the increasing concentration of $^{11}B_4C$ within the multilayer. The intention was to try to keep the same bilayer thickness for comparison, so the higher the $^{11}B_4C$ content the lower the Fe and Si content. Although the nominal bilayer thickness was 25 Å, with increasing $^{11}B_4C$ concentration the bilayer decreased in thickness, which is evident from the shift of the Bragg peak to higher scattering angles. However, this further solidifies the fact that the interface width decreased with higher amounts of $^{11}B_4C$ since the Bragg peak increased even though the intensity should decrease due to the Bragg peak appearing at higher scattering angles, according to Porod's law. Further, the addition of $^{11}B_4C$ in both layers decreases the X-ray SLD contrast, hence should in theory decrease the intensity of the Bragg peak with increasing concentrations of $^{11}B_4C$. However, apparent in Fig S1C, the Bragg peak still increased in intensity with higher concentrations, presenting evidence that the interface width decreased. However, too high concentrations of $^{11}B_4C$ could reduce the neutron polarization capabilities due to a decrease in magnetization. Thus 14 vol.% within the Fe layers was chosen to maintain sufficient magnetization while still being magnetically soft for the continuation of our study. Observing Fig S1D, the incorporation of $^{11}B_4C$ resulted in more intense Bragg peaks and higher orders of Bragg peaks for all measured bilayer thicknesses, supporting our idea of decreased interface width, regardless of bilayer thicknesses. The presence of Bragg peaks at higher Q-values enables the possibility of reflectivity at higher Q-regions. The figure also illustrates a Bragg peak for a bilayer thickness of 15 Å when $^{11}B_4C$ is present, whereas no Bragg peak is observed without $^{11}B_4C$. GenX3[21] was used to fit the Fe/Si and Fe/Si + $^{11}B_4C$ samples with 25 Å nominal bilayer

thickness, as shown in Fig. S1E. The analysis suggests that the Fe/Si sample contains 26 Å of heavily mixed Fe and Si atoms throughout the entire sample which suggests iron-silicides throughout the entire multilayer, however with approximately 8 Å Si rich areas resulting in the Bragg peak that we see in Fig. S1E. The addition of $^{11}B_4C$ prevented the formation of iron-silicide, resulting in a more clearly separated magnetic and non-magnetic layer, a decreased interface width, and increased reflectivity, along with an additional order Bragg peak.

Section S2. XPS and chemical bonding

Both Fe *2p* and Si 2p peaks shift towards higher binding energy for the $^{11}B_4C$ containing sample as compared to the Fe/Si sample. The shift is 0.65 eV and 0.45 eV, respectively. This can be explained by the formation of Fe-B, Fe-C, Si-B, and Si-C bonds. As both B and C are more electronegative than Fe and Si, the valence charge density on Fe and Si atoms is expected to decrease upon bonding. Thus, the hypothesis that the Fe-B bonds are what causes the amorphization is then confirmed.

Section S3. TEM and HAADF-STEM with EELS

From Fig. S3 it seems like the Fe/Si + $^{11}B_4C$ multilayer does not have an even distribution of B throughout the multilayer or within each layer. There seems to be a B-poor region within the Si layers. Combined with the results from the XPS, in Fig. S2, it is suggested that due to Si-C bonds within the Si layer the B would have a smaller probability to be deposited in Si rich regions.

Section S4. GIWAXS

Fig. S4A and Fig. S4B shows the grazing-incidence wide angle X-ray scattering (GIWAXS) of Fe/Si and Fe/Si + $^{11}B_4C$ multilayers, where Fig. S4A shows multilayers with a bilayer thickness of 100 Å and N = 10 and Fig. S4B multilayers with a bilayer thickness of 25 Å and N = 20. The GIWAXS data was taken simultaneously as the GISAXS measurements at the MiNaXS/P03 beamline using a LAMBDA 9M detector (X-Spectrum, pixel size = 55 µm) at a sample-to-detector distance of 284 mm. The background subtracted data is plotted using a temperature scale for intensity.

Grazing-incidence small-angle X-ray scattering (GIWAXS), Fig. S4A and Fig. S4B, shows the crystallinity of Fe/Si multilayers with various concentrations of $^{11}B_4C$. The quarter circles and ordered intensities stem from crystallinity within the samples known as Debye-Scherrer rings.

The intensity in the Fe/Si sample is concentrated on specific spots on the quarter circle, indicating a more defined ordering compared to a sample where the intensity is evenly distributed over the quarter circle. The broadness of the ring is related to the size of the crystallites. On the other hand, the GIWAXS pattern of the Fe/Si + $^{11}B_4C$ sample shows a broad and featureless subtle ring, indicating a lack of well-defined crystallographic planes in the material. However, it is interesting to note that the Fe/Si + $^{11}B_4C$ sample still retains this hint of the quarter circle seen in the Fe/Si sample. By adjusting the colormap intensity, it was possible to investigate a series of Fe/Si multilayers with varying concentrations of $^{11}B_4C$ to examine the transition from crystalline to amorphous in detail, as seen in Fig. S4(b). The intensity of the quarter circle shows a clear trend of going from well-defined and intense crystallinity to non-existent with increasing $^{11}B_4C$ concentration up to 40 vol.%. However, traces of crystallinity were detected up to 20 vol.% when compared to XRD results. This indicates that GIWAXS, with its higher sensitivity to crystallinity, was able to detect even small traces of crystallinity for higher $^{11}B_4C$ concentrations. Overall, the

results demonstrate the effectiveness of GIWAXS in identifying the presence of crystallinity in multilayer samples with varying concentrations of $^{11}B_4C$.

Section S5. Simulations of thicker bilayers using the extracted fitted parameters from Fig. 2.

Fig. S5 shows the simulated polarizing neutron reflectivity using the fitted parameters from Fig 2B. Fig. S5A represents the Fe/Si multilayer while Fig. S5B displays the Fe/Si + $^{11}B_4C$. It should be noted that the polarization results stated in the main text is related to the multilayers with the specified bilayer thicknesses and number of bilayers. For thicker bilayers, however, e.g. 200 Å, the polarization values are here predicted to not be substantially increased unless finer SLD tuning is performed.

Using the fitted parameters obtained from Fig. 2B, thicker bilayers were simulated, demonstrating that the polarization typically approaches 100%. Our study aimed to reflect and polarize at scattering angles/vectors that are not yet utilized or achievable by state-of-the-art neutron optics. Incorporating $^{11}B_4C$ into Fe/Si multilayers has been simulated to increase the polarization also for thicker bilayers as well although not as substantially as for thin bilayers where SLD matching is critical. The increase in polarization for a bilayer thickness of 200 Å was only 1%. Furthermore, it is theoretically possible to finetune the concentration of $^{11}B_4C$ to achieve even higher polarization, but this requires smaller increments of $^{11}B_4C$ to find the optimal amount for polarization enhancement.

Section S6. Elastic Recoil Detection Analysis (ERDA)

If the vol.% is a less desired measure, atomic percentages for all the samples obtained from elastic recoil detection analysis (ERDA) are presented in the tables below. Due to the depth resolution of ERDA being several nanometers and the samples of interest having layer thicknesses as small as a nanometer the following method was used to obtain the atomic percentages. All the samples measured with ERDA have the same amount of $^{11}B_4C$ in the Fe layer but various amounts in the Si layer. By measuring the amount of $^{11}B_4C$ in the whole multilayer of the Fe/Si + $^{11}B_4C$ (13.9 vol.%) sample and a sample with twice the amount of $^{11}B_4C$ in the Si layer but the same amount of $^{11}B_4C$ in the Fe layer the amount atomic pecentage of $^{11}B_4C$ within the Fe layer could then be calculated and using extrapolation also what percentages of $^{11}B_4C$ in Si the other samples should have. ERDA measurements on those other samples confirmed that the extrapolation were accurate with less than a percent's margin. Important to note however is that the atomic percentages within each layer is based on the volumetric percentages which in turn may not be very accurate as seen through XRR fits and TEM, due to the layer thicknesses differing from the nominal values.

ERDA measures the amount of each atom and since ^{11}B and C are not stoichiometrically 4:1 when deposited in films the atomic percentages show the total amount of ^{11}B atoms + C atoms. Another reason to count ^{11}B and C together is due to the fact that ^{11}B and C intensities appear too overlapped in the ToF-ERDA spectra to be able to separate them well. Although an approximate separation was made and can be seen in Table 3.

Tables 1, 2 and 3 shows the atomic compositions from ERDA of all multilayers. From Table 1 together with Fig. S1A we can conclude that by replacing every fifth Fe atom with a ^{11}B (or C) atom the Fe layer becomes X-ray amorphous and magnetically soft. From Table 2 along with Fig. 4C we obtain the atomic percentages of ^{11}B + C needed to achieve optimal polarization for 25 Å bilayer thickness and a thickness ratio of 0.5. However, since the thickness ratio turned out not to

be 0.5 the exact percentages may not be very accurate, although the relation between the samples are. From Table 3, it can be concluded that the actual composition of the incorporated $^{11}B_4C$ is closer to $^{11}B_{\sim5}C$.

Section S7. Experimental details for deposition system for samples from S1, S3 and Fig. S4B

Fe/Si and Fe/Si + $^{11}B_4C$ multilayer thin films were deposited using ion-assisted magnetron sputter deposition in a high vacuum system with a background pressure of about $5.6\cdot10^{-5}$ Pa ($4.2\cdot10^{-7}$ Torr). The multilayers were deposited onto 001-oriented single crystalline Si substrates, 10×10×1 mm^3 in size, with a native oxide. During deposition the substrate temperature was maintained at an ambient temperature (293 K), and to improve the thickness uniformity, the substrate was spinning at 8 rpm. The substrate table was electrically isolated, enabling a substrate bias voltage to be applied in order to attract sputter gas ions from the plasma. The main difference growing these samples compared to the other sample in this article was by using a modulated ion assistance regime during the deposition.[27] Ion-assisted deposition was employed by attracting Ar-ions from the sputter plasma through a negative substrate bias of -30 V. A magnetic field, colinear with the substrate normal, was used to condense the plasma towards the substrate, thereby increasing the Ar-ion flux at the substrate. The sputtering was modulated by alternating between 0 V substrate bias for the first approximately 3 Å to then employ the -30 V substrate bias for the rest of the layer. The modulated ion-assistance scheme is for the decrease in intermixing. The sputtering targets used were Fe (99.95% purity, 75 mm diameter), $^{11}B_4C$ (99.8% chemical purity, isotopic purity >90%, 50 mm diameter), and Si (99.95% purity, 75 mm diameter). The magnetrons were continuously running during deposition and the material fluxes were controlled using computer-controlled shutters placed in front of the magnetrons for each target material. This allowed for deposition of multilayers from the separate target materials, as well as the possibility of alloying two target materials to achieve a desired composition through co-sputtering. When depositing Fe/Si + $^{11}B_4C$, each bilayer consisted of co-sputtered $^{11}B_4C$ with Fe, followed by the deposition of $^{11}B_4C$ with Si. The deposition rates of both Fe + $^{11}B_4C$ and Si + $^{11}B_4C$ were nearly equal, approximately 0.5 Å/s. When preparing samples with different ratios of Si to $^{11}B_4C$, only the deposition rate of Si was adjusted by tuning its target power.

Figs. S1 to S5

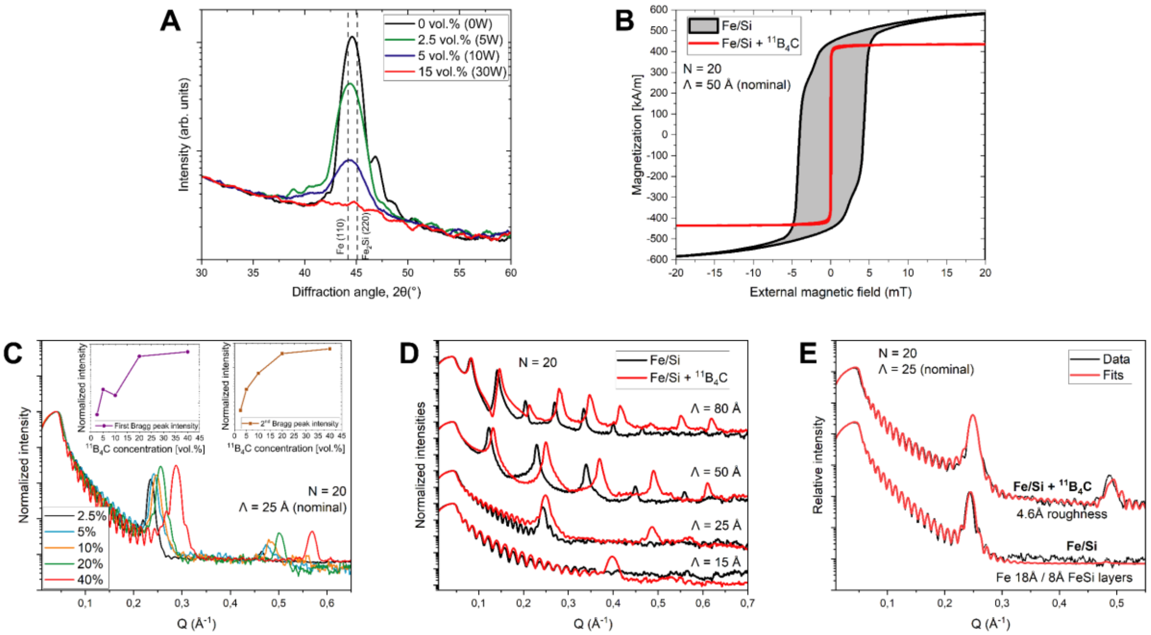

Fig. S1. Structure and magnetism. (**A**) X-ray diffraction (XRD) of 4 multilayers of Fe/Si with various volumetric percentages of $^{11}B_4C$ incorporated within the layers in the multilayer, ranging from 0 vol.% to 14 vol.%. The positions for metallic Fe 110 peak and the Fe_2Si silicide 220 peak are indicated. (**B**) Vibrating sample magnetometry (VSM) of a Fe/Si and a Fe/Si + $^{11}B_4C$ multilayer showing the hysteresis curves, where the grey area highlights the area within the magnetic coercivity. (**C**) X-ray reflectivity of Fe/Si + $^{11}B_4C$ samples with various ratios of $^{11}B_4C$ within the multilayers. The insets show the first (left) and second (right) order Bragg peak intensity as a function over the concentration of $^{11}B_4C$ within the multilayers. (**D**) X-ray reflectivity comparisons of Fe/Si and Fe/Si + $^{11}B_4C$ multilayers of various bilayer thicknesses, ranging from 15Å to 80 Å bilayer thickness. The curves have been stacked vertically for a clearer comparison between the material system (**E**) X-ray reflectivity data and fits of a Fe/Si and Fe/Si + $^{11}B_4C$ sample.

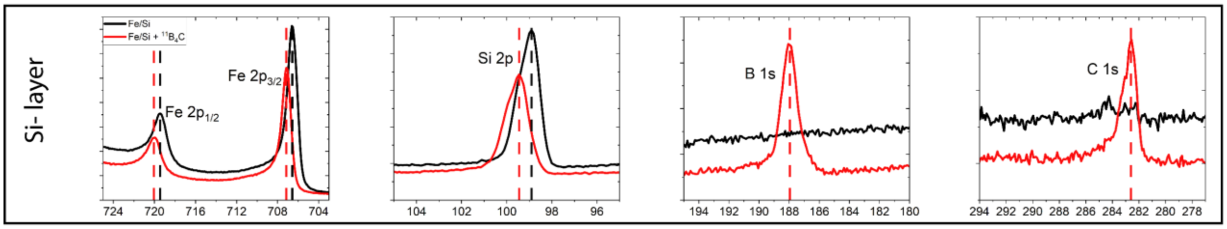

Fig. S2. Chemical bonds. XPS spectra from the Fe/Si (red) and Fe/Si + $^{11}B_4C$ (black) multilayer (N = 10, Γ = 0.5 (nominal) and Λ = 100 Å (nominal)), acquired within the top Si layer. The spectra from left to right Fe *2p*, Si *2p*, B *1s* and C *1s* respectively.

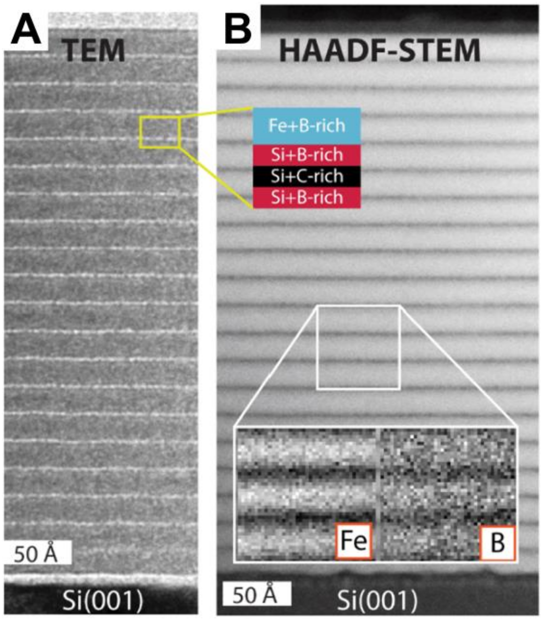

Fig. S3. Microscopy on the B and Fe out-of-plane spread. (**A**) Section of overview diffraction contrast TEM micrograph of Fe/Si + $^{11}B_4C$ multilayer with 14 vol.% of $^{11}B_4C$ from Fig. 4(D to F) and (**B**) corresponding scanning HAADF-STEM image and overlayed 150 x 150 Å^2 electron energy loss spectroscopy (EELS) map of Fe and B. From elemental analysis obtained using XPS, TEM, STEM, and EELS a schematic illustration of the elemental distribution in the multilayer stack is shown in the image.

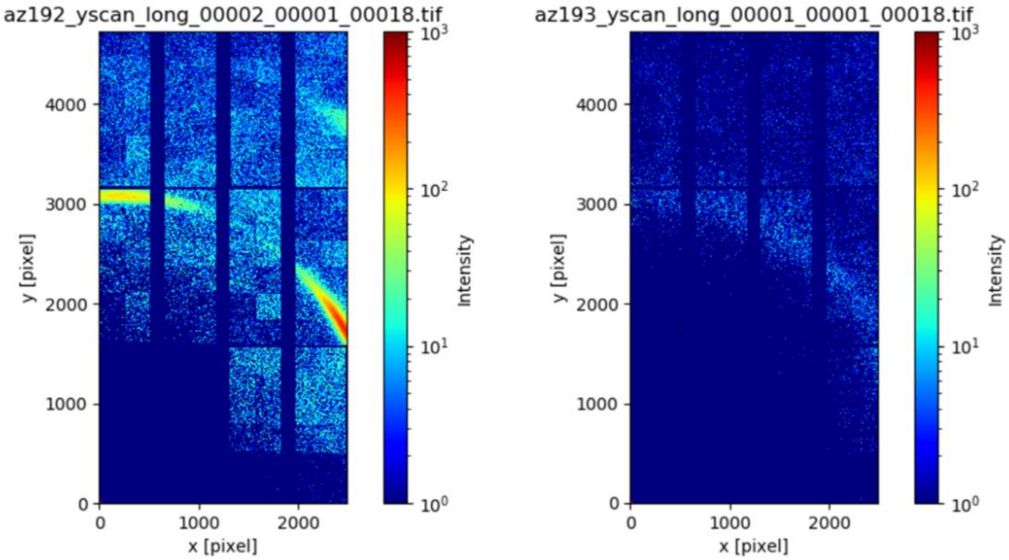

Fig. S4A. Crystallinity comparison. Grazing-incidence wide angle X-ray scattering (GIWAXS) patterns of Fe/Si (left) and Fe/Si + $^{11}B_4C$ (right) multilayers with a bilayer thickness of 100 Å and N = 10. The Fe/Si + $^{11}B_4C$ multilayer with a concentration of 14 vol.% of $^{11}B_4C$.

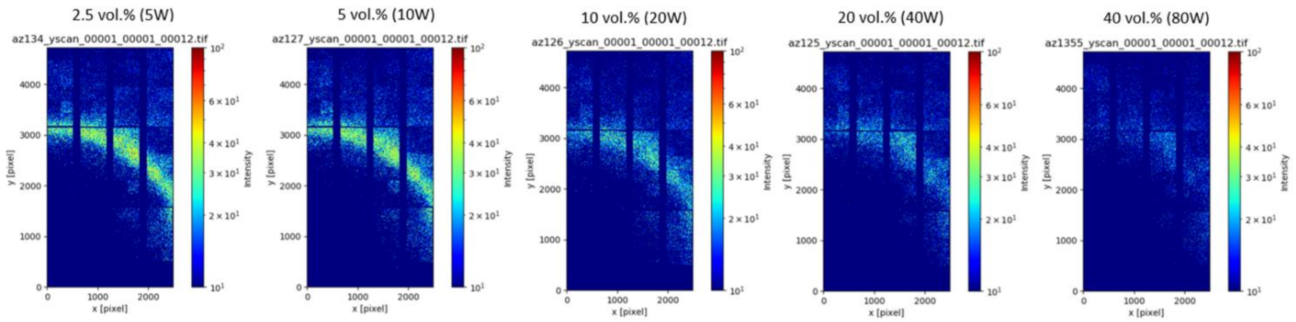

Fig. S4B. Transition from crystalline to amorphous structure with increasing $^{11}B_4C$ concentration. GIWAXS patterns of Fe/Si multilayers with varying concentrations of $^{11}B_4C$, with a fixed bilayer thickness of 25 Å and N = 20. Samples grown in a different chamber, see S7.

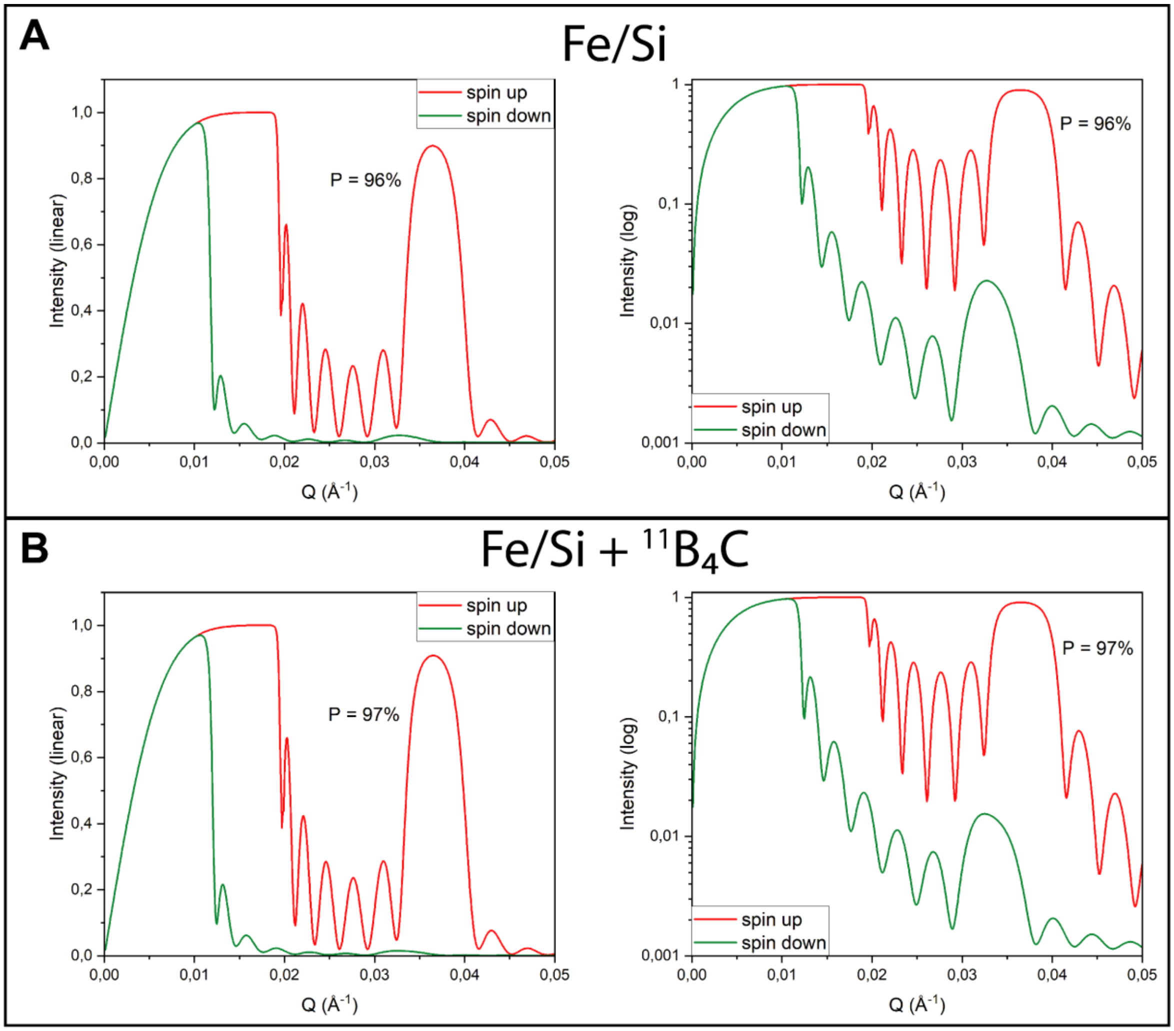

Fig. S5. Polarized neutron reflectivity (PNR) simulations. Using the fitted parameters from Fig. 2B. Linear intensity scale on left side and logarithmic on the right. (**A**) PNR for Fe/Si and (**B**) PNR for Fe/Si + $^{11}B_4C$ multilayers, both with Λ = 200 Å and N = 7.

Tables S1 to S3

Table S1. Atomic percentages of ^{11}B + C within the Fe layer for the various samples seen from the main text, determined using ERDA.

**Sample**	**^{11}B + C atomic percent within Fe layer**
All samples (averaged)	20 at.%

Table S2. Atomic percentages of ^{11}B + C within the Si layer for the various samples seen in Fig. 4, determined using ERDA.

**Sample**	**^{11}B + C atomic percent within Si layer**
Fe/Si	0.3 at.%
Fe/Si + $^{11}B_4C$ (13.9 vol.%)	15 at.%
Fe/Si + $^{11}B_4C$ (17.4 vol.%)	17 at.%
Fe/Si + $^{11}B_4C$ (24.0 vol.%)	19 at.%
Fe/Si + $^{11}B_4C$ (38.7 vol.%)	26 at.%

Table S3. Atomic percentage ratio between ^{11}B and C within throughout the entire multilayer for the various samples seen in Fig. 4, determined using ERDA.

**Sample**	**$^{11}B/C$ ratio throughout the entire multilayer**
Fe/Si	0.3
Fe/Si + $^{11}B_4C$ (13.9 vol.%)	4.9
Fe/Si + $^{11}B_4C$ (17.4 vol.%)	4.7
Fe/Si + $^{11}B_4C$ (24.0 vol.%)	4.9
Fe/Si + $^{11}B_4C$ (38.7 vol.%)	4.7
